
JOURNAL INFORMATION
==============================
Journal ID: Wirtschaftsdienst

Wirtschaftsdienst

EISSN: 1613-978X

ARTICLE INFORMATION
==============================
PMCID: PMC9017733
DOI: 10.1007/s10273-022-3165-9
Article ID: 3165
Article version: 1
Subjects: Ökonomische Trends

Inflation in Deutschland gewinnt an Fahrt

Inflation in Germany gains momentum

Berlemann Michael berlemann@hwwi.org
1
Eurich Marina marina.eurich@hsu-hh.de
2
Haustein Erik Erik.Haustein@hsu-hh.de
2
1 grid.435054.3 0000 0001 2227 8589 Hamburgisches WeltWirtschafts Institut gemeinnützige GmbH (HWWI), Oberhafenstraße 1, 20097 Hamburg, Deutschland
2 grid.49096.32 0000 0001 2238 0831 Fakultät für WiSo, Helmut-Schmidt-Universität/Universität der Bundeswehr Hamburg, Holstenhofweg 85, 22043 Hamburg, Deutschland
Electronic publication date: 2022 Apr 19
Print publication date: 2022
Volume: 102
Issue: 4
First page: 319
Last page: 320
Copyright: © Der/die Autor:in 2022
License: Open Access: Dieser Artikel wird unter der Creative Commons Namensnennung 4.0 International Lizenz veröffentlicht (https://creativecommons.org/licenses/by/4.0/deed.de).
Open Access wird durch die ZBW — Leibniz-Informationszentrum Wirtschaft gefördert.
License URL: https://creativecommons.org/licenses/by/4.0/

Keywords: E31, E52
issue-copyright-statement: © The Author(s) 2022

==============================

Am 30. März 2022 gab das Statistische Bundesamt bekannt, dass die Inflationsrate in Deutschland, gemessen an der Veränderung des Verbraucherpreisindex (VPI) zum Vorjahresmonat, im März um 7,3 % gestiegen ist (Statistisches Bundesamt, 2022, vgl. Abbildung 1). Damit erreicht die Inflationsrate einen Wert, der in Deutschland seit dem ersten Golfkrieg nicht mehr beobachtet wurde. Die Deutschen gelten als extrem inflationsavers (Berlemann und Enkelmann, 2013); insofern ist es keine Überraschung, dass die aktuelle Inflationsentwicklung große Sorgen in der Bevölkerung auslöst.

Im Nachgang der weltweiten Finanzkrise im Frühsommer 2007 und der darauffolgenden Weltwirtschaftskrise entwickelte sich Ende 2009 im Euroraum die Eurokrise (z. B. Lane, 2012). Als Folge der schlechten wirtschaftlichen Entwicklung und vieler ausfallender Wertpapiere kam das Interbanken-Kreditgeschäft im Euroraum weitgehend zum Erliegen und trocknete auch den Kreditmarkt für Unternehmen aus. Gleichzeitig kam es in mehreren südeuropäischen Staaten zu Problemen, Staatsanleihen an den Finanzmärkten zu platzieren und so die laufenden Staatsausgaben zu refinanzieren. Die EU beschloss daraufhin in Zusammenarbeit mit dem Internationalen Währungsfonds und der Europäischen Zentralbank (EZB) umfassende Stützungsmaßnahmen. Um den Kreditfluss zwischen den Banken und in den Unternehmenssektor zu befördern, senkte die EZB in mehreren Schritten den Hauptrefinanzierungssatz auf null. Zudem begann das Eurosystem mit einem Aufkauf von Staats- und auch Unternehmensanleihen über den Sekundärmarkt (z. B. Belke, 2010). Während diese Transaktionen anfänglich sterilisiert wurden, gab die EZB diese Neutralisierung 2014 auf. Auch wenn seitdem das Volumen der Anleihekäufe variierte, hielt die EZB seither im Grundsatz an dieser Politik fest und beließ den Hauptrefinanzierungssatz bei null. Trotz dieser ultraexpansiven Geldpolitik konnte eine Entkopplung der Konsumgüterinflation von der Geldpolitik beobachtet werden, was sich teilweise durch eine zunehmende Globalisierung der Wertschöpfungskette und einen steigenden internationalen Wettbewerbsdruck erklären lässt (z. B. Ascari und Fosso, 2021; Heise et al., 2022).

Das Ausbleiben hoher Inflationsraten bei Konsumgütern bedeutet jedoch nicht, dass die ultraexpansive Geldpolitik der EZB ohne Folgen geblieben wäre. Aufgrund der niedrigen Zinsen wurden andere Formen der Vermögensanlagen attraktiv. So haben sich in Deutschland seit Beginn der lockeren Geldpolitik nicht nur die Aktien-, sondern auch die Immobilienmärkte dynamisch entwickelt (vgl. Abbildung 2). Gleichzeitig reduzierte die Geldpolitik der EZB nicht nur für die südeuropäischen Staaten den ökonomischen Druck auf die Staatsfinanzen; auch der deutsche Fiskus profitierte von den niedrigen Zinsen. So ist der Anteil der Zins- an den Gesamtausgaben des deutschen Staates stark rückläufig und von 6,2 % (2007) auf 1,2 % (2020) gesunken (Eurostat, 2022).

Auslöser der aktuell zu beobachtenden Inflation war nicht die ultraexpansive Geldpolitik der EZB, sondern es waren vor allem die beiden jüngsten Krisenereignisse. Die seit zwei Jahren andauernde COVID-19-Pandemie sorgte für eine Unterbrechung der oft komplexen internationalen Lieferketten in der industriellen Produktion. Waren es zunächst die Eindämmungsmaßnahmen der Pandemie, die die Produktion und Lieferung von meist Vorprodukten erschwerte, ist es derzeit vorrangig der hohe Krankenstand im Zuge der hoch ansteckenden Omikron-Variante. Die Unterbrechung von Lieferketten sorgt dafür, dass trotz vorhandener Nachfrage die betroffenen Güter nicht oder nur in geringerer Menge produziert werden können. In der Konsequenz steigen die Preise der betroffenen Gütergruppen (z. B. Leibovici and Dunn, 2021). Der für 2022 erhoffte Erholungsprozess wurde durch den Angriff Russlands auf die Ukraine unterbrochen. Hier wurden nicht nur die über die Ukraine laufenden Lieferketten beeinträchtigt. Auch die strikte Sanktionspolitik des Westens gegen Russland führt zu Beeinträchtigungen im internationalen Handel.

Abbildung 1
**Deutsche Verbraucherpreise und EZB Zinssätze**
Monatswerte

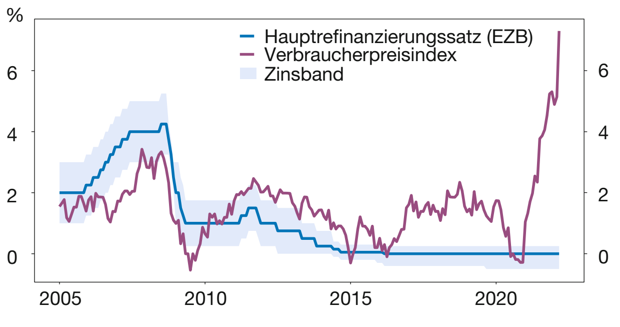

Quellen: Bundesbank und Statistisches Bundesamt (Destatis), 2022; eigene Darstellung.

Abbildung 2

**Vermögenspreise und EZB-Zinssätze**

durchschnittliche Quartalswerte

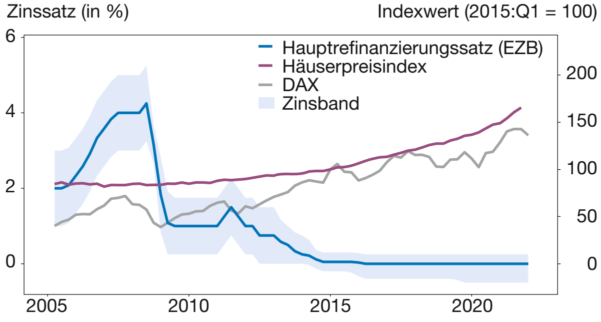

Quellen: Bundesbank, Macrobond und Statistisches Bundesamt (Destatis), 2022; eigene Darstellung.

Welche Produkte oder Dienstleistungen die derzeitige Inflationsrate am stärksten treiben, kann nur auf Basis disaggregierter Werte beurteilt werden. Inflationsraten nach Gütergruppen liegen für den März 2022 noch nicht vor, sodass auf die Februarwerte zurückgegriffen werden muss. Die höchste Preissteigerung wies im Februar 2022 Heizöl mit 40,0 % auf, gefolgt von Kraft- und Schmierstoffen für Kraftfahrzeuge (25,6 %), Gas (22,7 %) und festen Brennstoffen (19,0 %). Aber auch Lebensmittel gehören zu den Preistreibern (Speisefette und Speiseöle, 19,0 %; Gemüse, 10,1 %).

Da weder klar ist, wann die COVID-19-Pandemie oder der russische Invasionskrieg endet, ist momentan nicht abzusehen, zu welchem Zeitpunkt wieder mit ungestörten weltweiten Lieferketten gerechnet werden darf und somit der angebotsseitige inflationäre Druck abnehmen wird. Vielmehr steht zu befürchten, dass insbesondere die hohen Rohstoff- und Energiepreise auch Kostensteigerungen bei vielen anderen Produkten erzeugen und insofern zusätzlicher inflationärer Druck entstehen wird. Daher stellt sich die Frage, ob nicht eine deutlich restriktivere Geldpolitik angezeigt wäre, um die Inflationsraten wieder zu dämpfen. Restriktivere Refinanzierungskonditionen sind typischerweise dann sinnvoll, wenn Inflation nachfrageseitig verursacht wird, wie es in konjunkturellen Boomphasen der Fall ist. Die derzeitige Inflation ist aber weitgehend angebotsseitig verursacht und würde durch einen veränderten geldpolitischen Kurs gar nicht beseitigt. Gleichzeitig birgt eine restriktivere Geldpolitik die Gefahr, die Konjunktur abzuwürgen. Zudem würden stark steigende Zinsen vermutlich nicht ohne Effekte auf die Vermögensmärkte bleiben. Ein Einbruch der Aktienmärkte und der Immobilienpreise wird mit steigenden Zinsen wahrscheinlicher (Hanck und Prüser, 2020); welche Folgen solche Ereignisse haben können, ist spätestens seit der Subprime-Krise bekannt. Noch gravierender könnten die Effekte steigender Zinsen für die Staatsfinanzen werden. Durch die ultraexpansive Geldpolitik der EZB konnten viele EU-Staaten ihre massiven Haushaltsdefizite sehr günstig refinanzieren, sodass der Zinsdienst oftmals trotz stark steigender Verschuldung absolut sogar gesunken ist. Mit stark steigenden Zinsen und einem Auslaufen des Aufkaufs von Staatsanleihen durch die EZB dürften zahlreiche EU-Staaten schnell wieder Refinanzierungsschwierigkeiten erleben. Auch die jüngst im Zuge des russischen Überfalls auf die Ukraine ausgerufenen Ziele der Bundesregierung dürften unter einer wesentlich restriktiveren Geldpolitik viel schwerer zu erreichen sein.

Trotz der Risiken erscheint eine Änderung des geldpolitischen Kurses der EZB unausweichlich. Die Inflationserwartungen steigen bereits deutlich und drohen bald in eine Lohn-Preis-Spirale zu münden. Die Kosten einer Disinflationspolitik aus einer solchen Lohn-Preis-Spirale dürften viel höher als die eines frühzeitigen Gegensteuerns sein (z. B. Filardo, 1998). Eine schnelle Reaktion der EZB könnte die Inflationserwartungen kurzfristig wieder senken und eine Lohn-Preis-Spirale vermeiden. Eine Strategieanpassung ist ohnehin unausweichlich, da die EZB früher oder später auf die striktere Geldpolitik der Federal Reserve Bank reagieren muss, um massive Kapitalabflüsse zu vermeiden. Nach einer langen Phase der ultraexpansiven Geldpolitik wäre bereits die glaubhafte Erklärung der Abkehr von dieser Praxis ein starkes Signal für die Märkte, sodass auch kleine Zinsschritte künftig ausreichen könnten, die Inflationserwartungen zu stabilisieren. Tatsächlich erklärte die EZB am 10. März 2022, aus den Anleihekäufen im Sommer 2022 aussteigen zu wollen und danach eine sanfte Anhebung der Zinsen zu erwägen (EZB, 2022). Ob ein so langsamer Strategiewechsel allerdings ausreichen wird, darf bezweifelt werden.

Michael Berlemann, Marina Eurich, Erik Haustein
HWWI, Helmut-Schmidt-Universität


Literatur

Ascari, G. und L. Fosso (2021), The Inflation Rate Disconnect Puzzle: On the International Component of Trend Inflation and the Flattening of the Phillips Curve, De Nederlandsche Bank Working Paper, 733.
Belke A Driven by the markets? ECB sovereign bond purchases and the securities markets programme Intereconomics 2010 45 6 357 363 10.1007/s10272-010-0356-1
Berlemann M Enkelmann S Die „German Angst“ — Inflationsaversion in Ost- und Westdeutschland ifo Dresden berichtet 2013 2 3 9
Europäische Zentralbank (2022), Geldpolitische Beschlüsse, https://www.ecb.europa.eu/press/pr/date/2022/html/ecb.mp220310∼2d19f8ba60.de.html (4. April 2022).
Filardo, A. J. (1998), New Evidence on the Output Costs of Fighting Inflation, Federal Reserve Bank of Kansas City Economic Review, 33–61.
Hanck C Prüser J House prices and interest rates: Bayesian evidence from Germany Applied Economics 2020 52 28 3073 3089 10.1080/00036846.2019.1705242
Heise S Karahan F Şahin A The Missing Inflation Puzzle: The Role of the Wage-Price Pass-Through Journal of Money, Credit and Banking 2022 54 7 51 10.1111/jmcb.12896
Lane P The European Sovereign Debt Crisis Journal of Economic Perspectives 2012 26 3 49 68 10.1257/jep.26.3.49
Leibovici, F. und J. Dunn (2021), Suppy Chain Bottlenecks and Inflation: The Role of Semiconductors, Economic Synopses, 28.
Statistisches Bundesamt (2022), Inflationsrate im März 2022 voraussichtlich +7,3%, Pressemitteilung, 137, https://www.destatis.de/DE/Presse/Pressemitteilungen/2022/03/PD22_137_611.html (4. April 2022).
